# Exploring the Effect of Dehydration on Water Migrating Property and Protein Changes of Large Yellow Croaker (*Pseudosciaena crocea*) during Frozen Storage

**DOI:** 10.3390/foods10040784

**Published:** 2021-04-06

**Authors:** Mingtang Tan, Jing Xie

**Affiliations:** 1 College of Food Science and Technology, Shanghai Ocean University, Shanghai 201306, China; mingtang120@163.com; 2 Shanghai Engineering Research Center of Aquatic Product Processing & Preservation, Shanghai 201306, China; 3 Professional Technology Service Platform on Cold Chain Equipment Performance and Energy Saving Evaluation, Shanghai 201306, China; 4 National Experimental Teaching Demonstration Center for Food Science and Engineering, Shanghai 201306, China

**Keywords:** large yellow croaker (*Pseudosciaena crocea*), ice crystal, dehydration, water migrating property, protein changes

## Abstract

This study aimed to explore the effect of dehydration on the water migrating property and protein changes of large yellow croaker during frozen storage. A freeze-dryer was used to accelerate experiments, which was isolated from oxygen and excluded the effects of protein oxidation. After dehydration time (3, 9, 18, and 30 h) for both fast- and slow-freezing samples, the results showed that the ice sublimation of samples containing small ice crystals was faster than that of samples containing large ice crystals in the early stages of dehydration, but in the latest stage, there was an opposite trend. The results indicated that dehydration reduced the water freedom degrees and water–protein interaction. At the same time, dehydration had a significant effect on protein secondary and tertiary structures. The significant increase in surface hydrophobicity and particle size indicated that dehydration exacerbated myofibrillar protein aggregation. The ΔH1 values (from 1.275 to 0.834 J/g for slow-freezing group and from 1.129 to 0.855 J/g for fast-freezing group) decreased gradually as the dehydration time extended, indicating the decrease in protein thermal stability. Additionally, significant protein degradation occurred when the water content of the sample decreased to a certain level. This study showed that ice crystal size had an important effect on the rate of ice sublimation, and the occurrence of dehydration during frozen storage accelerated the water loss and the decrease in protein stability.

## 1. Introduction

Freezing may cause a variety of adverse effects on aquatic products, such as freeze concentration, mechanical damage, freezer burn, and recrystallization [1]. It is usually accompanied by a decrease in quality, including oxidation of proteins and lipids, deterioration of texture, and a decrease in water content [2]. The formation of large ice crystals and uneven distribution in aquatic products tissue can cause poor organoleptic quality and loss of nutrients, such as deterioration of drip loss and softening [3]. In particular, product dehydration during frozen storage leads to weight loss and the loss of valuable nutrients which might result in the detriment of both businesses and consumers.

The majority of water is trapped in the protein-dense networks of muscle structure, which accounts for about 65% to 80% of muscle tissue [4]. When the ice vapor pressure on the surface of the product is lower than that of the surrounding air during frozen storage, the ice sublimates from the surface of the product to its surrounding environment, resulting in dehydration, which is also known as freezer burn if the dehydration is serious [5]. This phenomenon can occur from the surface of the product to its core. The water migration and the interaction between water and protein of lamb meat during 35 °C air drying were investigated by Rao et al. [6]. The results indicated that bound water, which was tightly bound to protein macromolecules (T_2b_), and immobilized water that was located in the myofibrillar protein network (T_21_) played an important role in the water migration of lamb during air drying, and besides, the denaturation of myosin may lead to water migration from myofibrils to the space between myofibrils. Likewise, the decrease in water content of the sample due to water evaporation could also result in protein denaturation [6]. Thus, there is a strong relationship between the water content in frozen foods and its degree of protein denaturation. In addition to the occurrence of dehydration during frozen storage, there is also ice recrystallization [3] and protein oxidation and denaturation [7] caused by frozen storage, making it impossible to directly prove the specific effect of dehydration on protein denaturation.

Freeze drying is a dehydration process by direct sublimation of ice crystals from a frozen product [8]. In order to investigate the effect of ice crystal morphology on the efficiency of dehydration, Jin et al. [9] added biogenic ice nucleation proteins (INPs) to sucrose solution in a freeze-dryer, and the results indicated that the ice crystals formed by INPs were larger in size and had lamellar structure, which was less obstructive to water vapor flow and more conducive to the occurrence of dehydration of frozen products. On the contrary, the literature suggests that in frozen muscle tissue, small crystals with a higher surface free energy and thermodynamic instability are more potent in melting than large crystals at lower temperatures [10,11]. The evidence for this relationship between ice crystals size and dehydration remains inconclusive. Consequently, it is necessary to evaluate the effect of ice crystal size on dehydration. Further, we wished to confirm the effect of dehydration on the water migrating property and protein changes of samples while excluding the changes such as protein oxidation and ice recrystallization caused by prolonged frozen storage. The significant difference in the size of ice crystals formed by refrigerator freezer and spiral freezer had been confirmed in a previous experiment.

For this purpose, this work used a freeze-dryer to accelerate the ice sublimation of large yellow croakers during frozen storage. Samples containing different ice crystal sizes were observed to determine their water migrating property and their protein changes, and an attempt was made to describe the relationship between dehydration and protein denaturation. The purpose of this study was to explore the mechanism of how product quality is affected by dehydration during frozen storage and to understand the effect of ice crystal size on dehydration of aquatic products, which will help to slow down quality degradation due to dehydration during frozen storage of aquatic products.

## 2. Materials and Methods

### 2.1. Sample Processing

Live large yellow croakers (*Pseudosciaena crocea*) (480–500/g, about 30 cm in length) purchased from a seafood port in Shanghai, China, were shipped to the laboratory and then wrapped in ice to death. The remaining surface water, blood, and slime on the samples were dried by paper towel. Before being assigned to one of the two freezing treatments, namely slow freezing (−18 °C refrigerator) and fast freezing (spiral freezer with 6.67 m/s wind speed), the samples were packed in sealed bags to prevent the water loss of samples during freezing due to the wind speed. When the core temperature of these samples reached −18 °C, the frozen samples removed from the sealed bags were subsequently loaded on the shelf of a freeze-dryer to simulate the crystalized ice sublimation of large yellow croakers during frozen storage.

In this study, the parameters adopted for the freeze-dryer were as follows: (1) the vacuum in the drying chamber was controlled at 200–300 ubar, (2) the temperature of the cold trap was −40 °C, and (3) the shelf temperature was controlled at −18 °C. The fast-freezing samples were placed in a freeze-dryer for 3, 9, 18, and 30 h and were named F3, F9, F18, and F30, respectively. Similarly, these slow-freezing samples treated by the freeze-dryer were named S3, S9, S18, and S30. Subsequently, all samples were transferred to vacuum bags for maximum isolation from surrounding air. Finally, samples were stored in a −80 °C freezer (to reduce the effect of ice recrystallization), and all testing was completed within two weeks. The back muscle of the fish was used for the determination of all indicators.

### 2.2. Measurement of Weight Loss and Water Content

The thawed sample muscle was weighed before dehydration treatment (*W*_1_), and the sample was weighed again at the end of treatment (*W*_2_). Weight loss was calculated as shown in Equation (1):

(1) $$\mathrm{Weight}\;\mathrm{loss}/\%=\frac{W_{1}-W_{2}}{W_{1}}\times 100$$

The water content of large yellow croakers was assessed with a water tester (HX-Q10, Shanghai Huxi Analytical Instrument Factory Co., Ltd., Shanghai, China). Five grams of thawed sample muscle (*W*_3_) was heated at 105 °C until the weight change was less than 0.001 g in 1 min; then, the sample was removed and the water content (g of water per 100 g of sample) was calculated according to the difference in mass before and after (*W*_4_) drying as shown in Equation (2):

(2) $$\mathrm{Water}\;\mathrm{content}/\frac{g}{100g}=\frac{W_{3}-W_{4}}{W_{3}}\times 100$$

### 2.3. Measurement of Unfrozen Water Content

The unfrozen water (UFW) content was determined using a differential scanning calorimeter (DSC-8500, PerkinElmer, Norwalk, USA) according to the method of Li et al. [12] with some modifications. About 15 mg of the frozen sample muscle was placed in a tightly sealed aluminum pan. It was left at a starting temperature of −40 °C for 2 min and then heated to a final temperature of 35 °C at a rate of 10 °C/min. The UFW content (g/100g) was calculated as follows:

(3) $$\mathrm{UFW}=\left(W-\frac{\Delta H}{\Delta H_{0}}\times 100\right)$$

where Δ*H* is the heat of fusion for the samples, J/g; Δ*H*_0_ indicates the heat of fusion for pure water at 0 °C, 333.88 J/g; and *W* is the water content in the sample, which was determined using the initial method, g/100 g.

### 2.4. Low-Field Nuclear Magnetic Resonance (LF-NMR) and Magnetic Resonance Imaging (MRI)

The water distribution and states of large yellow croakers were determined by a MesoMR23-060H.I LF-NMR analyzer (Niumag Corporation, Shanghai, China) according to a previously described method [13,14]. Thawed samples (2.0 cm × 2.0 cm × 1.0 cm) were taken from the dorsal muscles of the croaker, dried to remove surface water, and wrapped with fresh-keeping film. Each sample was examined in a 70 mm diameter cylindrical probe with a working temperature of 32 °C. Acquisition parameters were as follows: receiver bandwidth frequency (SW) = 100 kHz; analog gain (RG1) = 20.0 dp; P1 = 19.00 μs; digital gain (DRG1) = 6; TD = 400,068; preamplifier gain (PRG) = 1; duration between successive scans (TW) = 2000 ms; number of the scans (NS) = 4; P2 = 38.00 μs; time echo (TE) = 0.500 ms; and NECH = 8000. The MRI imaging software and MSE spin-echo imaging sequence acquisition were used to generate the proton density weighted images of the samples.

### 2.5. Total Sulfhydryl (SH) Content

To extract myofibrillar proteins (MFPs) from fish, 2 g of thawed croaker muscle was homogenized in 20 mL of buffer A (50 mmol/L KCl, 20 mmol/L Tris-maleate, pH 7.0) using a homogenizer, followed by centrifugation at 10,000× *g* for 15 min at 4 °C. The protein precipitate was then repeatedly centrifuged as in the previous step. Finally, the obtained precipitate was mixed with 20 mL of buffer B (0.6 mol/L KCl, 20 mmol/L Tris-maleate, pH 7.0), homogenized, extracted at 4 °C for 3 h, and then centrifuged at 10,000× *g* for 15 min at 4 °C. The obtained supernatant was the MFP solution required for this experiment.

The total SH content of the MFP was measured using the total SH measurement kit (Nanjing Jiancheng Bioengineering Institute, Nanjing, China), and results were expressed as μmol/g prot.

### 2.6. Determination of MFP Aggregation

#### 2.6.1. Surface Hydrophobicity

The surface hydrophobicity (S_0_-ANS) of MFP was determined using ANS (8-anilino-1-naphthalene sulfonate) as a fluorescence probe according to Nian et al. [15]. The MFP was diluted to 0.2, 0.4, 0.8, and 1.0 mg/mL protein solution with 20 mM PBS (pH 6.7, 0.6/M NaCl). Subsequently, 4 mL protein solutions were each added to 20 μL 20 mM PBS (containing 8 mM ANS, pH 7.0) and allowed to stand for 10 min at room temperature (25 °C) under dark conditions. S_o_-ANS of each sample was acquired from the slope of the relative fluorescence intensity of the solution measured with an F-7100 fluorescence spectrophotometer (Hitachi Co., Tokyo, Japan) at an excitation wavelength of 390 nm, an emission wavelength of 470 nm, and a slit width of 10/nm.

#### 2.6.2. Particle Size

The particle size of MFP was measured using a Mastersizer 3000 (Malvern Instruments Ltd., Malvern, UK). The material refractive index and absorption coefficient were set to 1.46 and 0.001, respectively.

### 2.7. Protein Secondary Structure

Raman spectrometer (HR800, Horiba/Jobin. Yvon, Longjumeau, France) was used to analyze the changes of protein secondary structure following the method of Zhang et al. [16]. The thawed samples were transferred to a glass slide for measurement. The parameters were as follows: excitation wavelength of 532 nm, scanning range 400–3800 cm^−1^, instrument power of 100 mW, resolution of 2 cm^−1^, exposure time of 60 s, slit width of 200/μm, raster of 600/g/mm, and number of sample scans of 3. The spectral data were baseline corrected, smoothed, and normalized using Omnic 8.0 software (Thermal Fisher Scientific, Madison, WI, USA). Peakfit 4.12 software was used to quantify the relative content of protein secondary structure.

### 2.8. Protein Tertiary Structure

The tertiary structure of MFP solutions (0.05 mg/mL) was measured by an F-7100 fluorescence spectrophotometer. The parameters were as follows: excitation wavelength of 295/nm, excitation and emission slit widths of 5 nm, emission spectra range 300–410/nm, scanning speed of 1200/nm/min, and PMT voltage of 400 V.

### 2.9. Protein Thermal Stability Properties

Changes in thermal stability of proteins were measured using a differential scanning calorimeter (TA Q2000, TA Instruments, New Castle, DE, USA) as previously described by Liu et al. [17] with slight modifications. Thawed samples (10 mg) were accurately weighed and placed in a tightly sealed aluminum pan, while a sealed empty aluminum pan was used as a control group. Samples were equilibrated at 20 °C for 2 min and then heated from 20 to 100 °C at a heating rate of 5 °C/min.

### 2.10. Sodium Dodecyl Sulfate–Polyacrylamide Gel Electrophoresis (SDS-PAGE)

SDS-PAGE of MFP was performed as described by Wang et al. [18]. SDS-PAGE was performed continuously on a pre-cast 12% Hepes–Tris gel, and protein standards ranging from 11 to 245 kDa purchased from EZBiolab were used to determine protein molecular weight after electrophoresis.

### 2.11. Statistical Analysis

In this study, all measurements were repeated in triplicate. The results were expressed as mean ± standard deviation. One-way ANOVA analysis and Duncan’s test were performed on the experimental data using SPSS 25.0 (SPSS Inc., Chicago, IL, USA), and *p* < 0.05 indicated that the means differed significantly. Pearson correlation was used to calculate linear correlations of variables between unfrozen water content and bound water, and figures were created with Origin 2018 software (OriginLab Corporation, Northampton, MA, USA).

## 3. Results and Discussion

### 3.1. Weight Loss and Water Content Analysis

**Figure 1:**
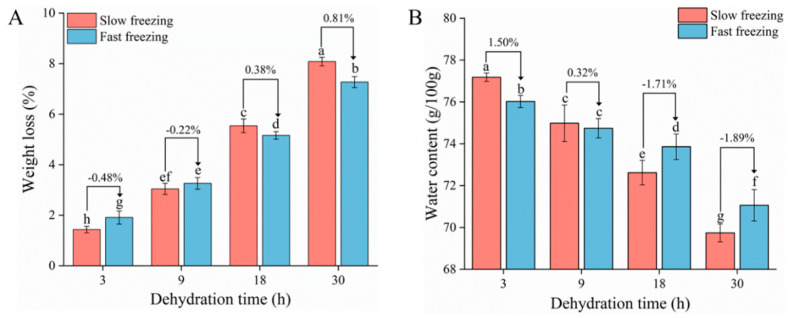
Effect of dehydration treatment on weight loss (**A**) and water content (**B**) of large yellow croaker. Different lowercase letters in the same index indicate significant differences (*p* < 0.05). The water content of the fresh sample (CK) was 83 ± 0.63%.

**Figure 2:**
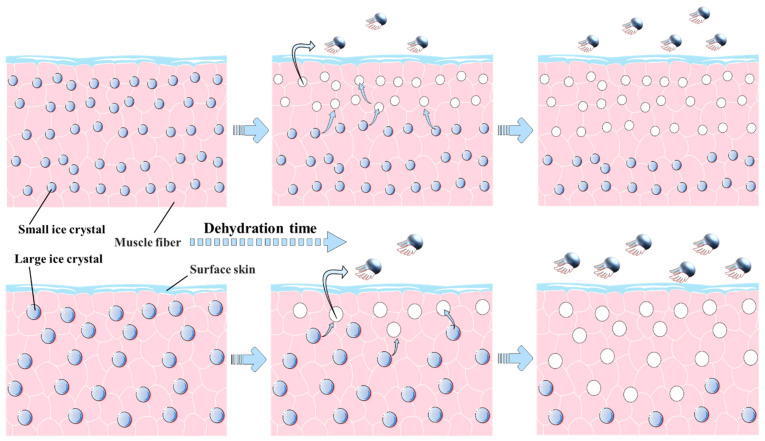
Schematic diagram of ice crystal sublimation during dehydration.

The ice sublimation can be mimicked by a freeze-dryer, and Figure 1 shows the weight loss and water content of thawed large yellow croaker at different dehydration times. The weight loss of the slow-freezing group decreased more slowly than that of the fast-freezing group during the first 9 h, but in the later stage, the slow-freezing group had more weight loss than the fast-freezing group. The same trend was also shown for the water content. The water content of the S3 group was 1.50% higher than that of the F3 group, while the water content of the S30 group was 1.89% lower than that of the F30 group. Compared to large ice crystals, small ice crystals with higher thermodynamic energy are more capable of sublimation at low temperatures [19]; hence, samples containing small ice crystals are more liable to cause weight loss and decrease in water content at the initial time of dehydration (Figure 2). Smaller pores were left after sublimation of small ice crystals on the sample surface; however, they are more resistant to the subsequent water vapor flow during sublimation [20]. Based on this, larger ice crystals can instead exacerbate the water loss of the sample during prolonged dehydration. It is also shown in Figure 1 that the rate of water loss gradually decreased for all samples with the extension of dehydration time, and ice crystals inside the samples covered by the surface muscles were probably not susceptible to sublimation due to the increasing mass transfer resistance. Therefore, it is essential to suppress water loss at the initial stage of frozen storage in order to obtain a higher quality product.

### 3.2. Water Status Analysis

**Figure 3:**
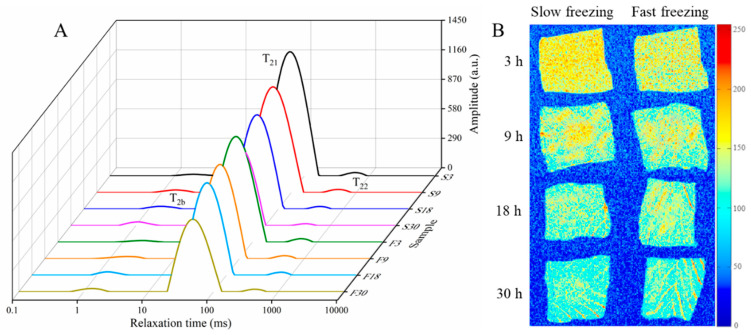
Transverse relaxation time (T_2_) curves (**A**) and MRI pseudocolor images (**B**) of thawed large yellow croakers during dehydration.

There are three water states in thawed croaker: bound water (T_2b_), immobilized water (T_21_), and free water (T_22_). In Figure 3A, it can be seen that the majority of the water in these samples was immobilized water, and as the water content of samples decreased, its peak area (A_21_) gradually decreased and the peak position migrated to the left, which indicated that the freedom degrees of immobilized water gradually decreased and the water was more tightly bound to non-water components [21,22]. The possible reason was ascribed to the water loss with higher freedom degrees in immobilized water during dehydration. The T_2_ relaxation time and peak area of the three water populations are shown in Table 1. Similarly, it was found that the decrease in water content led to a decrease in T_2b_, T_22_, and A_22_ during dehydration. However, the A_2b_ of the bound water showed an increasing trend, which was because the relaxation area, as a relative area, decreased for the other two kinds of water. Similar results were also obtained in the drying experiments of shrimp [22] and abalone [23]. The reduction of bound water, which is tightly bound to protein macromolecules, may contribute to the degradation and denaturation of muscle proteins.

The results in Table 1 indicate a decreasing trend in the unfrozen water (UFW) content as the water content of the samples decreased. The UFW in frozen aquatic products is mainly bound water that is closely attached to the polar groups on the surface of protein macromolecules [12]. Therefore, changes in bound water content due to dehydration could reflect the aggregation and conformational changes of proteins during this period [24]. In addition, the decline in UFW content occurred mainly in the late stage of dehydration.

Figure 3B shows the changes in the water distribution of croaker samples during dehydration. The grayscale values from high to low are indicated by a color scale from red to blue, respectively. A gradual decrease in the bright region of all samples could be clearly observed with the dehydration process, which meant that the water was continuously removed from the fish. There was an obvious phenomenon of uneven intensity of water in the sample for the first 9 h, which may be due to the sublimation of ice crystals on the surface of the sample first, followed by the internal water vapor transfer to the sample surface during dehydration. It is worth noting that the water content of samples containing small ice crystals showed poor results in the first 9 h compared to those recorded for large ice crystals, but it had better results in the later stages, which is also observed in Section 3.1.

### 3.3. Protein Oxidation

Changes in protein structure expose sulfhydryl (SH) groups buried inside the protein, leading to the oxidation of SH groups, their transformation into disulfide groups, and a decrease in SH content [25]. According to the results of total SH content in Table 1, the dehydration treatment did not have a significant effect on the total SH content of myofibrillar proteins (MFP). This could be attributed to the fact that the samples were dehydrated under vacuum and isolated from oxygen, resulting in the inability of the SH group to be oxidized to disulfide bonds. The total SH content was found to slightly decrease in the late stage of dehydration. The aggregation occurring after protein unfolding is due to hydrophobic interactions, at which the distance between SH groups is shorted and the groups connect to form disulfide bonds, leading to a decrease in the total SH content of MFP [26]. Alternatively, it was suggested that masking of SH groups caused by protein aggregation results in a reduction in detectable total SH groups [27]. Therefore, the use of a freeze-dryer to freeze samples represents an approximate method to exclude the effects of protein oxidation caused by prolonged frozen storage.

### 3.4. Protein Secondary Structure

**Figure 4:**
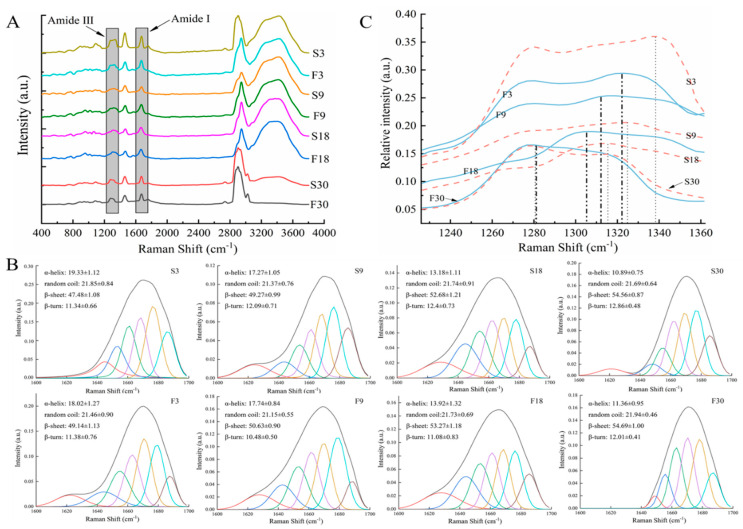
Effect of dehydration treatment on Raman spectra (**A**), iterative curve-fitted (**B**), and amide III region of Raman spectra (**C**) of thawed large yellow croakers.

The wave number and intensity of the Raman spectrum can be used to monitor the changes of protein secondary structure [25]. The Raman spectra of large yellow croaker muscle (400–3800 cm^−1^) treated with smooth and baseline calibration are presented in Figure 4A. The amide bonds of proteins had several different modes of vibration, where the protein secondary structure (α-helix, random coil, β-sheet, and β-turn) is mainly investigated by amide I (1600–1700 cm^−1^) [16,28]. The amide I region was selected for deconvolution and second-order derivatives, and iterative curve fitting yielded information on individual Raman peaks (Figure 4B).

The contents of α-helix (1650–1658 cm^−1^), random coil (1660–1665 cm^−1^), β-sheet (1665–1680 cm^−1^), and β-turn (1680–1690 cm^−1^) could be determined from the amount of area under the peak as shown in Figure 4B [24]. Notably, β-sheet was the main protein secondary structure of samples during dehydration. The α-helix content decreased from 19.33% to 10.89% and 18.02% to 11.36% for the slow- and fast-freezing groups. The β-sheet increased from 47.48% to 54.56% and 49.14% to 54.69% for the slow- and fast-freezing groups, respectively. However, the random coil and β-turn contents did not change significantly. This implies that dehydration may mainly lead to the unfolding of α-helical structures and the conversion to β-sheet. The decrease in the content of α-helical structures also meant that more hydrophobic groups were exposed due to the dehydration of sample, resulting in an increasing surface hydrophobicity [21]. Zhang et al. [16] suggested that the stability of α-helix and β-sheet depend on the stability of hydrogen bonds within and between peptide chains, respectively. The increased β-sheet content implies an increase in hydrogen bonding, leading to enhanced protein–protein interaction [29]. Therefore, the occurrence of dehydration may lead to the breakage of intramolecular hydrogen bonds of the peptide chain, followed by the enhancement of intermolecular hydrogen bonds between peptide chains, resulting in protein aggregation. In addition, the changes in protein secondary structure at the late stage of dehydration were our focus to maintain the quality of aquatic products.

The amide III band (1230–1350 cm^−1^) is mainly generated by the CN stretching of the peptide bond coupled to NH in-plane bending vibrations, which can be used to reflect changes in the protein secondary structure [30]. Figure 4C clearly shows that the relative peak intensity of the highest Raman peak gradually decreased and the corresponding peak position moved toward the small wavenumber as the ice crystals continued to sublimate during dehydration. A decrease in the α-helix content of fish protein was found to reveal a more intense band and a shorter wavelength [25]. Thus, dehydration caused a decrease in the α-helix content of protein and a decrease in the stability of protein secondary conformation, which was consistent with the results obtained by amide I band analysis.

### 3.5. Protein Tertiary Structure

**Figure 5:**
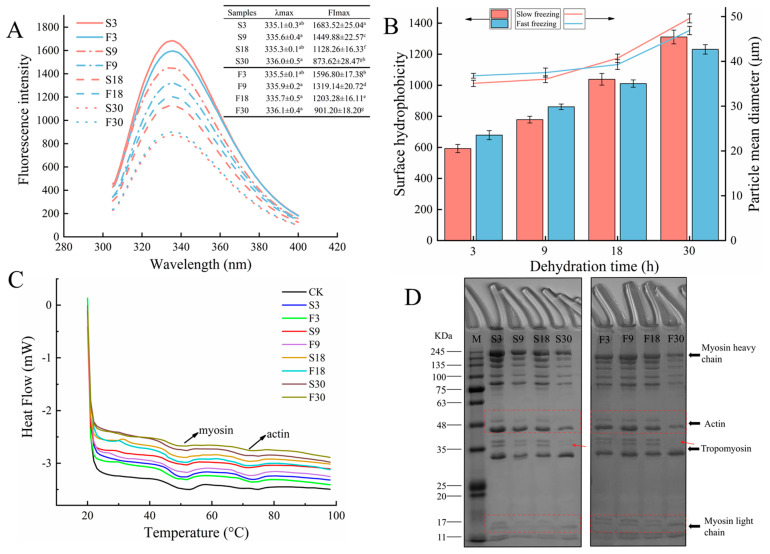
Effect of dehydration treatment on intrinsic fluorescence spectra (**A**), surface hydrophobicity and particle size (**B**), differential scanning calorimetry (**C**), and SDS-PAGE (**D**) of large yellow croakers. Different lowercase letters in the same index indicate significant differences (*p* < 0.05).

The changes in the protein tertiary structure can be evaluated by the number intensity of tryptophan residues and its polarity of the microenvironment measured by endogenous fluorescence spectroscopy [31]. The maximum fluorescence emission wavelength value of the fresh sample was 333.4 ± 0.5. When tryptophan residues buried in a hydrophobic microenvironment were exposed to a polar environment, the more hydrophilic microenvironment caused protein unfolding, resulting in a redshift (longer wavelengths) in the maximum fluorescence emission wavelength (λ_max_). Nevertheless, the results presented in Figure 5A show no significant difference in the λ_max_ of fluorescence peak (*p* > 0.05). This suggests that the dehydration treatment could not enhance the polar environment around the increased tryptophan residues. In terms of tryptophan fluorescence intensity, the maximum fluorescence intensity (FI_max_) decreased remarkably (*p* < 0.05) with increasing dehydration time of the samples, in the order of S3, F3, S9, F9, F18, S18, F30, and S30. When tryptophan residues are mostly embedded in the core of the protein, there is a higher fluorescence intensity [32]. Thus, the action of dehydration led to protein tertiary structure unfolding. Due to the exposure of hydrophobic groups after protein unfolding, proteins tend to aggregate with each other via hydrophobic interactions [31]. This result was verified in the following for surface hydrophobicity and particle size distribution.

### 3.6. MFP Aggregation

#### 3.6.1. Surface Hydrophobicity

The surface hydrophobicity (S_0_-ANS) of proteins reflects the content of hydrophobic amino acid residues on the protein surface and is capable of characterizing the changes in protein conformation [33]. The S_0_-ANS value of the fresh sample was 316.48 ± 36.11. The effect of dehydration on the S_0_-ANS of myofibrillar proteins in large yellow croaker is depicted in Figure 5B. The gradual increase in S_0_-ANS values of the fast-freezing and slow-freezing groups with the increase in the dehydration time was noticeable, indicating that ice sublimation promoted myofibrillar protein unfolding and thus exposed more hydrophobic groups. Hence, dehydration induced a change in the conformation of the protein molecule. Of note, more S_0_-ANS was shown in the fast-freezing group compared to the slow-freezing group during the first 9 h of ice sublimation, while the opposite trend was observed in the later period. This was the same trend as for the water content, and it could be assumed that the dehydration of samples caused the changes in the protein molecule conformation. Zhang et al. [34] reported an increase in protein hydrophobicity in shrimp during frozen storage and attributed it to protein unfolding as well as the exposure of hydrophobic aliphatic and aromatic amino acids. The hydrophobic amino acid residues originally embedded inside the protein molecule were exposed to the protein surface, and these residues, through hydrophobic interactions, led to the onset of protein aggregation and a decrease in S_0_-ANS [35]. However, the S_0_-ANS values here showed a constant increase, indicating that the myofibrillar protein unfolding and aggregation had not yet reached equilibrium. The α-helix of the myosin tail contains a large number of hydrophobic residues [36]. Therefore, the content of the α-helix decreased after a disruption, and more hydrophobic residues were exposed, which could further verify the protein secondary structure analysis.

#### 3.6.2. Particle Size

The mean particle diameter of myofibrillar proteins was used here to assess the myofibrillar protein aggregation induced by dehydration (Figure 5B). The particle size value of the fresh sample was 10.41 ± 0.38 μm. The results suggested that the dehydration treatment exhibited significant differences (*p* < 0.05) in the particle mean diameters of MFP with increasing treatment time. Precisely, the observed increase in particle size after the dehydration treatment occurred mainly in the later stages of treatment. The increase in protein particle size may be due to protein aggregation caused by the oxidation and denaturation of MFP [37]. Cai et al. [32] also reported that dehydration tended to form protein aggregates. When dehydration reduces water availability, muscle proteins are exposed to solutes with higher ionic strength, and this process may lead to modifications in protein structure as evidenced by the formation of new covalent bonds and protein aggregation [4].

### 3.7. Protein Thermal Stability

Protein thermal stability is an important parameter in evaluating fish protein stability. As seen in Figure 5C, DSC thermograms of croaker muscle had two endothermic peaks, peaks 1 and 2, which appeared near 50 °C and 75 °C and represented myosin and actin, respectively [17]. The denaturation temperature (T_max_) and denaturation enthalpy (ΔH) of protein showed a negative correlation with the degree of protein denaturation. According to Table 2, the T_max_1 of samples decreased significantly (*p* < 0.05) with increasing dehydration time, but the change of T_max_2 was not significant (*p* > 0.05), indicating that dehydration mainly caused irreversible denaturation of myosin, while actin was more stable. In addition, Rao et al. [6] found that denaturation of myosin was an important cause of water migration from the interior of lamb muscle fibers to the exterior. A decrease in ΔH means that less energy is required to denature the protein and that the protein has become less stable. ΔH1 decreased from 1.275 to 0.834 J/g in the slow-freezing group, while it decreased from 1.129 to 0.855 J/g in the fast-freezing group; therefore, dehydration led to a reduction in energy necessary to resist myosin denaturation and a decrease in myosin stability, especially for samples containing large ice crystals in the later stages of dehydration. The decrease in the protein thermal stability may be a result of the breakage of hydrogen bonds between protein molecules caused by water loss, resulting in the degradation of protein structure and the formation of protein aggregation [26,38].

### 3.8. SDS-PAGE Analyses

SDS-PAGE was used to characterize the effect of dehydration treatment on the degradation of MFP by determining the molecular weight (Figure 5D). The appearance of myosin heavy chains (MHC, bout 200 kDa) may be due to cross-linking and aggregation of myofibril proteins, and the appearance of myosin light chains (MLC, 14–21 kDa) may be caused by the degradation of heavy chains [39]. As shown in Figure 5D, the protein molecules of the croaker samples frozen and dehydrated for 18 h did not change significantly. The samples containing large or small ice crystals, nevertheless, showed the disappearance of actin (45 kDa), tropomyosin (38 kDa), and MLC bands after 30 h of dehydration, which indicated the appearance of breaks in protein covalent bonds and primary structure [16]. Simultaneously, there was a slight decrease in the intensity of the MLC band, probably due to protein degradation and fragmentation or aggregation caused by the high activity of histoproteases [25]. The results of SDS-PAGE indicated that significant protein degradation occurred when the water content of the sample decreased to a certain level.

## 4. Conclusions

This study effectively explored the effects of dehydration on water migrating property and protein changes in large yellow croaker during frozen storage. A freeze-dryer was to accelerate experiments, isolate samples from oxygen, and exclude the effects of protein oxidation and ice recrystallization during frozen storage. Based on the variation of sample weight loss and water content, the results indicated that the ice sublimation of samples containing small ice crystals was faster in the early stage of dehydration compared to that of samples containing large ice crystals, while the water loss was hindered in the later stage because of the resistance of small pore size to water vapor flow. The significant decrease in T_2_ values indicated that water mobility gradually decreased as the water content of samples decreased. Meanwhile, the decrease in bound water content caused by dehydration reflects the aggregation and conformational changes of proteins. However, the total SH content showed only a slight decrease. As determined by Raman spectrometry and intrinsic fluorescence, a decrease in α-helix content and fluorescence intensity and an increase in β-sheet content were induced by dehydration, demonstrating that protein secondary and tertiary structures were destroyed during the dehydration process. Increasing surface hydrophobicity and particle size indicated that ice sublimation caused exposure of the hydrophobic groups and aggregation of myofibrillar proteins during dehydration. Additionally, the results of DSC and SDS-PAGE revealed that dehydration led to a decrease in thermal stability of proteins and an increase in MFP degradation, especially irreversible denaturation of myosin. Therefore, avoiding dehydration is important to maintain the quality of large yellow croakers during frozen storage. This study provides a theoretical basis for delaying the dehydration of aquatic products during frozen storage. In addition, temperature fluctuations are inevitable during frozen storage and further understanding of their effect on the dehydration rate is important.

## Figures and Tables

**Table 1:** NMR parameters, unfrozen water content, and total SH content of large yellow croakers during dehydration.

Sample	Bound Water		Immobilized Water		Free Water		UFW (g/100g)	Total SH Content (μmol/g)
	T_2b_(ms)	A_2b_(1/g)	T_21_(ms)	A_21_(1/g)	T_22_(ms)	A_22_(1/g)		
S3	1.96 ± 0.13 ^a^	257.11 ± 7.98 ^g^	54.79 ± 2.53 ^a^	21,341.01 ± 78.38 ^a^	622.26 ± 74.66 ^a^	313.22 ± 16.36 ^ab^	31.62 ± 0.78 ^a^	18.15 ± 0.26 ^a^
S9	1.96 ± 0.12 ^a^	343.46 ± 4.86 ^f^	51.11 ± 0.49 ^b^	19,166.92 ± 67.84 ^b^	541.59 ± 57.91 ^b^	293.20 ± 9.10 ^c^	28.46 ± 0.52 ^c^	18.27 ± 0.23 ^a^
S18	1.48 ± 0.12 ^bc^	385.73 ± 6.19 ^cd^	47.69 ± 1.28 ^c^	15,224.69 ± 59.93 ^ef^	439.76 ± 30.80 ^d^	231.89 ± 7.47 ^e^	23.99 ± 0.33 ^e^	17.98 ± 0.21 ^ab^
S30	1.12 ± 0.14 ^cd^	459.91 ± 4.50 ^a^	41.50 ± 0.80 ^d^	11,908.50 ± 61.07 ^h^	410.27 ± 52.63 ^e^	164.98 ± 11.98 ^g^	19.10 ± 0.63 ^g^	16.84 ± 0.17 ^a^
F3	1.96 ± 0.10 ^a^	246.29 ± 2.71 ^h^	54.79 ± 2.44 ^a^	19,077.90 ± 62.31 ^bc^	580.52 ± 61.45 ^ab^	337.16 ± 17.29 ^a^	29.08 ± 0.46 ^b^	18.22 ± 0.25 ^a^
F9	1.70 ± 0.21 ^ab^	371.81 ± 4.74 ^e^	51.11 ± 0.99 ^b^	17,113.04 ± 65.26 ^d^	505.26 ± 28.38 ^c^	279.88 ± 13.14 ^cd^	28.53 ± 0.29 ^bc^	18.03 ± 0.20 ^a^
F18	1.59 ± 0.12 ^b^	394.41 ± 4.33 ^c^	51.11 ± 1.56 ^ab^	16,077.90 ± 83.30 ^e^	471.38 ± 81.33 ^cd^	263.37 ± 20.26 ^d^	26.75 ± 0.57 ^d^	17.81 ± 0.24 ^b^
F30	1.29 ± 0.11 ^c^	428.12 ± 5.18 ^b^	47.69 ± 3.27 ^c^	12,327.77 ± 107.5 ^g^	439.76 ± 101.26 ^d^	220.42 ± 28.53 ^ef^	21.04 ± 0.70 ^f^	17.05 ± 0.33 ^c^

Values are mean ± standard deviation. Different lowercase letters in the same line indicate significant differences (*p* < 0.05).

**Table 2:** T_max_ and ΔH values obtained of myosin and actin of large yellow croaker muscle during dehydration.

Sample	Myosin		Actin	
	T_max_1 (°C)	∆H1 (J/g)	T_max_2 (°C)	∆H2 (J/g)
CK	54.04 ± 0.45 ^a^	1.611 ± 0.01 ^a^	74.52 ± 0.30 ^a^	0.506 ± 0.014 ^b^
S3	49.94 ± 0.25 ^b^	1.275 ± 0.012 ^b^	71.75 ± 0.23 ^b^	0.550 ± 0.012 ^ab^
S9	48.80 ± 0.18 ^d^	1.122 ± 0.012 ^c^	71.10 ± 0.18 ^bc^	0.572 ± 0.004 ^a^
S18	48.10 ± 0.30 ^e^	0.947 ± 0.008 ^e^	70.59 ± 0.75 ^c^	0.498 ± 0.006 ^b^
S30	47.26 ± 0.41 ^f^	0.834 ± 0.014 ^gh^	70.72 ± 0.16 ^c^	0.517 ± 0.012 ^b^
F3	49.42 ± 0.13 ^c^	1.129 ± 0.009 ^c^	71.35 ± 0.20 ^b^	0.564 ± 0.008 ^ab^
F9	48.31 ± 0.49 ^de^	0.968 ± 0.010 ^d^	71.47 ± 0.16 ^b^	0.535 ± 0.014 ^b^
F18	48.05 ± 0.24 ^e^	0.923 ± 0.006 ^f^	71.23 ± 0.42 ^b^	0.472 ± 0.010 ^c^
F30	47.68 ± 0.19 ^f^	0.855 ± 0.013 ^e^	71.51 ± 0.35 ^b^	0.485 ± 0.003 ^c^

Values are mean ± standard deviation. Different lowercase letters in the same line indicate significant differences (*p* < 0.05).

## Data Availability

All relevant data are included in the article.
